# Feasibility and Usability of a Digital Perinatal Navigator for High-Risk Pregnancies: Mixed Methods Study

**DOI:** 10.2196/88015

**Published:** 2026-10-08

**Authors:** Michelle Foerstel, Kristina Killinger, Mitho Mueller, Verena Engel, Katrin Schlobohm, Anna Sophie Scholz, Maren Goetz, Armin Bauer, Markus Wallwiener, Michael Abou-Dakn, Dorothea Scholle, Ekkehard Schleussner, Stephanie Wallwiener

**Affiliations:** 1 Department of Gynecology and Obstetrics University Hospital Heidelberg Heidelberg, Baden-Wurttemberg Germany; 2 Department of Psychology Ludwig-Maximilians-Universität München München Germany; 3 Institute for Communication and Assessment Research Heidelberg Germany; 4 Institute Women’s Health GmbH Tübingen Germany; 5 Department of Pediatric and Adolescent Medicine University Hospital Heidelberg Heidelberg, AL Germany; 6 Department of Gynecology University Hospital Halle Halle Germany; 7 Department of Obstetrics and Gynecology St. Joseph's Hospital Berlin Berlin Germany; 8 Department of Obstetrics Jena University Hospital Jena Germany; 9 Department of Obstetrics and Perinatal Medicine University Hospital Halle Halle Germany

**Keywords:** feasibility, health service accessibility, mHealth, mobile health, patient-centered care, perinatal care, perinatal mental health

## Abstract

**Background:**

The journey to parenthood involves significant physical, emotional, and psychosocial changes. Mental health challenges impact both maternal and fetal health, potentially leading to obstetric complications and developmental risks for children. Access to needed perinatal support is often limited due to individual and structural barriers. Digital health solutions can offer opportunities to provide low-threshold, personalized, and scalable support. We developed a digital navigator offering personalized guidance and connecting users to relevant support services with interactive follow-ups to self-assess their well-being. However, evidence regarding the feasibility of digital solutions in high-risk patients is limited.

**Objective:**

This study aimed to assess the feasibility and usability of a digital perinatal navigator app designed to provide personalized support and connect individuals with high-risk pregnancies to relevant health and social services.

**Methods:**

Conducted at University Women’s Hospital Heidelberg, the study used convenience sampling for a 2-week app test phase. A convergent mixed methods design integrated qualitative interviews (n=30) with psychometric surveys (n=35). Analyses included thematic analysis of interviews, descriptive statistics, 2-tailed paired *t* tests, and Pearson correlations. Results were triangulated at the end to better understand barriers to use.

**Results:**

Participants (median age 33 years; median gestational age 30 weeks) reported moderate to high rates of stress, anxiety, and depressive symptoms. Usability ratings were excellent (median System Usability Scale [SUS] score 80, IQR 17.5; median mHealth App Usability Questionnaire [MAUQ] score 111, IQR 28). Knowledge of health service providers increased significantly (mean +1.2 points; *P*<.01), with modest improvements in use. Qualitative analysis revealed key success factors such as intuitive structure, trustworthy medical content, and personalized information. Technical disruptions, navigation challenges, limited personalization, and incomplete regional integration of health care services were reported as barriers.

**Conclusions:**

The results indicate high feasibility and usability for our digital navigator in this high-risk population. The identified barriers are to be considered in the further development of the app and other perinatal digital care programs.

## Introduction

### Overview

The journey to parenthood involves significant physical, emotional, and psychosocial changes. Pregnancy is associated with a variety of challenges, and the quality of life of pregnant women can be influenced by sociodemographic, psychological, and physical factors. Symptoms of mental health disorders, obstetric complications, and pregnancy-related symptoms, such as nausea, are all linked to a poorer quality of life [1]. Postnatal depression and parental stress also affect daily life and partnerships. Parents frequently report feelings of inadequacy, and a lack of joy can contribute to feelings of shame and guilt in mothers [2].

Mental health challenges are a significant concern during pregnancy, with research indicating that depression affects approximately 9.3% of pregnant individuals, anxiety impacts 16.9%, and the prevalence of at least 1 mental health disorder reaches as high as 43.6% [3]. These conditions can significantly impact both maternal and fetal health. For example, negative emotions and poor mother-infant bonding are associated with the development of postnatal depression [4]. Depression during pregnancy is also linked to an increased risk of complications, such as preterm birth, low birth weight, or intrauterine growth restriction, and can negatively affect the unborn child [5]. Prenatal depression and anxiety are particularly detrimental to the socioemotional development of children [6]. Additionally, maternal psychological distress is associated with poorer developmental outcomes during the school years [7]. Medical conditions such as prematurity, gestational diabetes, and preeclampsia can also negatively affect child development [8-10]. In turn, depression is associated with other medical conditions, such as hypertension, preeclampsia, or gestational diabetes [5,11-13]. Especially multiple risks in early years lead to a disadvantage in children’s cognitive and behavioral development [14,15].

Nevertheless, numerous evidence-based support mechanisms exist to mitigate these challenges. Early detection of potential risks and intensified perinatal care can play a crucial role in protecting mothers and children from potential complications. For example, targeted interventions can improve the socioemotional development of children [16]. Additionally, early child interventions lead to substantial cognitive, behavioral, health, and schooling benefits [17,18]. Optimal care not only helps to manage complications effectively but can also minimize psychological symptoms, ease parental stress, and strengthen the mother-child bond [19-21]. These findings highlight the critical need for targeted interventions and support to address the mental health needs of expectant parents.

### The Role of eHealth in Perinatal Care

eHealth refers to the use of digital technologies in health care [22]. Numerous digital interventions, for example, in the form of telemedicine, have proven effective in treating pregnancy-related conditions such as postpartum depression [19] and have been shown to improve overall obstetric outcomes [19,23]. App-based interventions, specifically those targeting mental health issues such as depression or substance abuse during pregnancy, have also demonstrated effectiveness [24-26]. Recent reviews suggest that the implementation of eHealth can improve antenatal care access and use while being cost-effective in a time of financial constraints on health care systems [27,28].

### Barriers to Accessing Support Services

In Germany, the nationwide “Frühe Hilfen” (early help) program offers support for families with children aged 0 to 3 years, providing low-threshold access to services based on individual needs [29]. The KiD 0-3 study showed that knowledge of pregnancy counseling centers and family midwives is not reduced among less educated parents. This is attributed to secured funding and higher rates of offers for child welfare services [30]. An extended postnatal home visiting program in Rinkeby (Sweden) also showed potential for positive effects for parents and children [31]. However, access to support programs remains uneven. The KiD 0-3 study found that parents with lower educational levels are less likely to be aware of or use services such as family counseling, early support, or online consultation services [30].

The KUNO-Kids study showed that good knowledge of antenatal and perinatal support services is linked to higher education, no migration background, and better health literacy. In contrast, knowledge was lower among first-time mothers and unmarried women [32].

Similarly, high psychosocial stress and migration background further limit the knowledge of these services [30,32]. Moreover, in families facing multiple risk factors, it is often difficult to identify suitable support services and coordinate the necessary help [14].

This highlights the need for low-threshold navigators—tools that help inform families about available support options and facilitate access to relevant services. As outlined in the “Final Report of the Working Group on Children of Mentally Ill Parents” (Abschlussbericht der AG Kinder psychisch kranker Eltern), there remains a significant demand for resources that can guide families through the complex network of support services [33].

Our app-based navigator (called Mamaherz [Mum’s heart]) seeks to address this gap by offering a needs-based solution that connects users to relevant advice and support centers. In developing the app (Figure 1), we involved key stakeholders, including parents, pregnant women, health care providers such as midwives, regional health service providers (HSPs), and doctors [34].

**Figure 1:**
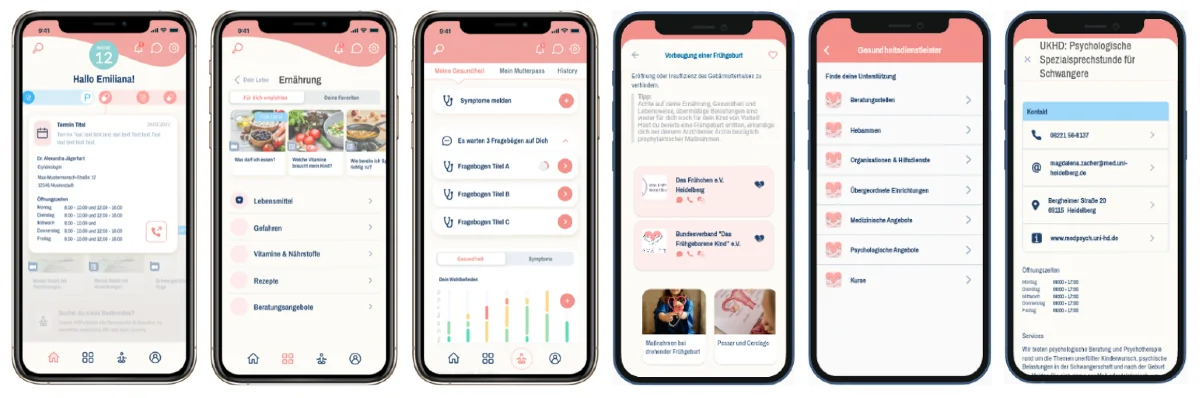
Representative user interface screens of the app-based navigator.

### Research Aim

The primary aim of this pilot study was to assess the feasibility and usability of the Family eNav pregnancy navigator app in a real-world obstetric setting. Feasibility was defined by recruitment retention, completion of the test phase, and interviews; usability was assessed using the System Usability Scale (SUS) and the mobile app rating questionnaire for users (mHealth App Usability Questionnaire [MAUQ]), as well as through interviews.

Secondary objectives were to characterize the psychosocial burden of the study cohort, including levels of depressive symptoms, anxiety, perceived stress, and pregnancy-related anxiety, as well as to evaluate changes in knowledge and use of HSPs after a 2-week app exposure. Specifically, we sought to determine whether the app could be effectively implemented for families with high-risk pregnancies defined according to established obstetric criteria, including women with preexisting conditions such as hypertension, diabetes, or mental health disorders (eg, depression or anxiety), as well as those experiencing pregnancy complications like preterm birth risk, intrauterine growth restriction, or gestational diabetes.

In addition, the study explored barriers and success factors related to achieving high acceptance of the app. Based on the findings, the app was adapted and optimized to better meet user needs.

## Methods

### Ethical Considerations

The study was approved by the Ethics Committee of the University of Heidelberg (S-344/2022). Study enrollment and informed consent were either given after personal recruitment from our study or through the project website. Potential participants were given the opportunity to enroll in the study online. For this purpose, the pregnant women were redirected to a separate page that comprehensively presented the goals and procedures of the project. The participation requirements were checked online through short questions that had to be answered; the study description as well as the study information and consent form had to be downloaded. Once this was done, the participant received an access code to unlock the app after downloading it. All participant data were deidentified prior to analysis. Direct identifiers were removed and replaced with unique study codes. Data were stored on encrypted, secure servers at University Hospital Heidelberg in compliance with the General Data Protection Regulation. Only the research team had access to the data, and all team members completed training in data protection and confidentiality. Any published results present only aggregated data, ensuring that individual participants cannot be identified. No financial compensation was provided to participants.

### Study Design

We decided on a mixed methods, parallel convergent study design to explore user experiences alongside psychometric outcomes and measures of knowledge and use. During interpretation, the authors compared quantitative and qualitative findings across the 2 components of the study and integrated them iteratively to identify converging, complementary, and explanatory insights. For example, when survey results showed high interest in app-based support and interview data revealed barriers such as lack of orientation and information overload, these findings were synthesized to refine the overall interpretation of user needs and app design requirements [35]. The research complied with the GRAMMS (Good Reporting of a Mixed Methods Study) guidelines [36] (Multimedia Appendix 1).

### Recruitment

We used convenience sampling based on patient availability and willingness to participate. Participants who met the inclusion criteria (Textbox 1) were approached through the antenatal outpatient clinic and obstetric ward at the University of Heidelberg, a level 1 perinatal center with around 2400 births per year and high numbers of extreme premature births. They were invited to take part in the study after having a short conversation with our recruiters concerning their availability and interest and self-reported pregnancy risk factors. Patients included in the study tested the perinatal guide for 2 weeks.

**Inclusion and exclusion criteria for study participation.**

**Inclusion criteria**

- Aged ≥18 years
- Intact pregnancy at 12-34 weeks’ gestation
- Fluent in German
- Internet-enabled smartphone
- Inpatient or outpatient treatment at the Department of Gynecology and Obstetrics, University Hospital Heidelberg
- Willingness to test the Mamaherz app
- Written consent for study participation

**Exclusion criteria**

- Aged <18 years
- No pregnancy, <12 weeks’ gestation, or >34 weeks’ gestation
- Limited German proficiency
- No access to an internet-enabled smartphone
- No treatment at the Department of Gynecology and Obstetrics, University Hospital Heidelberg
- Unwillingness to test the app
- No written consent for study participation

The perinatal guide includes personalized care plans, providing tailored content based on individual needs. Users have access to self-education tools, a variety of HSPs, and personalized information about available support services. Additionally, the app allows users to track symptoms and regularly complete short questionnaires about their well-being. The aim was to create an easily accessible app that supports pregnant women and parents during early parenthood, with a focus on regional services.

Our aim was to recruit approximately 50 participants between September and December 2023 for our pilot of the Family eNav project, which has an overall target of 500 participants and officially started recruitment in December 2023. Family eNav is a multicenter randomized controlled trial (RCT) assessing the efficacy of a perinatal app-based health and social service program. Testing the guide in a high-risk cohort gave us the opportunity to evaluate the individualization of care plans and their feasibility in a more condensed form in comparison to a random sample.

### Quantitative Data Collection and Analysis

Participants filled out electronic questionnaires in the app portal on the first day and at the end of the test phase after day 14 (Figure 2). Questionnaires were provided to participants in the same manner as planned for the RCT to gain a clear understanding of their psychometric profile and to test whether the data collection was feasible. Completion of questionnaires was not mandatory to use the app, but reminders were sent to increase participation. At the outset, each patient completed an onboarding questionnaire, which included sociodemographic information and patient history to estimate their risk profile. We defined 4 risk profiles: psychological, medical, preterm, and psychosocial. When the answers in the questionnaire indicated a particular risk, the user was assigned to the corresponding care plan. Users could also be assigned to multiple care plans.

The Questionnaire on Knowledge and Utilization of Health Service Providers (Q-HSP) was customized from the survey by Eickhorst et al [30], covering additional support domains (eg, pregnancy counseling, family/parenting counseling, and parent-child groups). Responses were provided on a 5-point Likert scale (knowledge: “no knowledge” to “extensive”; use: “not used” to “highly used”). The questionnaire allows binary classification (no knowledge vs knowledge; not used vs used) and calculation of individual mean knowledge and use scores (Multimedia Appendix 2). The questionnaire was validated in a German cohort [32].

The German version of the Edinburgh Postnatal Depression Scale (EPDS) assesses depressive symptoms over the past 7 days (prenatal/postnatal) [37,38]. It consists of 10 items, with 0 to 3 points each (total 0-30; no symptoms=0 and max burden=30). It was used descriptively and correlated with HSP knowledge and use (Cronbach α=0.81).

The State-Trait Anxiety Inventory (STAI) consists of two 20-item scales: STAI state scale (STAI-S; temporary/state anxiety) and STAI trait scale (STAI-T; dispositional/trait anxiety) [39]. Responses are rated on a Likert scale from 1 to 4 (total 20-80). It was used descriptively and correlated with HSP outcomes (Cronbach α=0.90).

**Figure 2:**
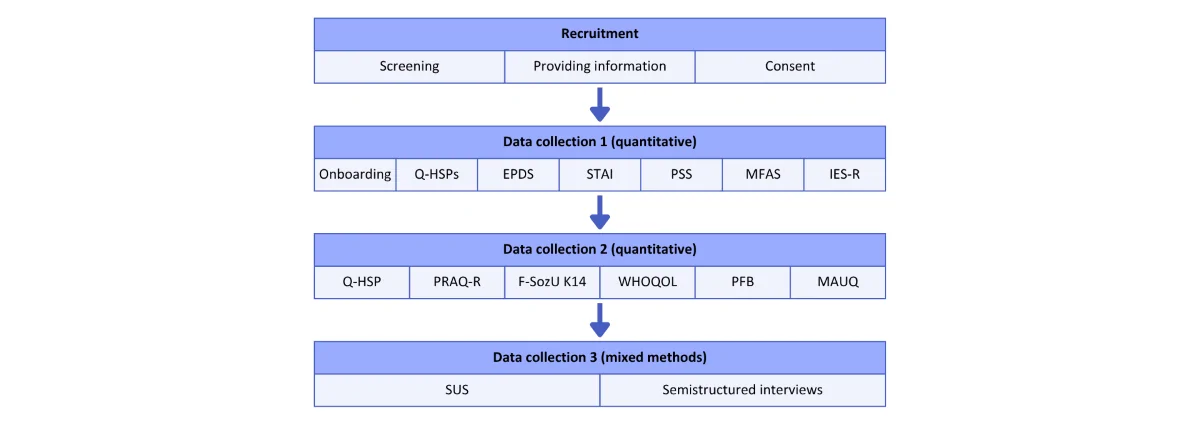
Study design of the 2-week pilot study: mixed methods, parallel convergent approach. EPDS: Edinburgh Postnatal Depression Scale; F-SozU: Social Support Questionnaire; IES-R: Impact of Event Scale-Revised; MAUQ: mHealth App Usability Questionnaire; MFAS: Maternal Fetal Attachment Scale; PFB: Partnership Questionnaire; PRAQ-R: Pregnancy-Related Anxiety Questionnaire-Revised; PSS: Perceived Stress Scale; Q-HSP: Questionnaire on Knowledge and Utilization of Health Service Providers; STAI: State-Trait Anxiety Inventory; SUS: System Usability Scale; WHOQOL: World Health Organization Quality of Life.

The German version of the Perceived Stress Scale (PSS) assesses stress over the past month [40]. It consists of 2 subscales: perceived helplessness (6 items) and perceived self-efficacy (4 items), rated on a 5-point Likert scale (higher scores = higher stress; Cronbach α =0.78).

The German version of the Maternal Fetal Attachment Scale (MFAS) assesses prenatal bonding [41]. It consists of 24 items, with 1 to 7 points each (total 24-168; higher score = stronger bond; Cronbach α=0.79).

The Impact of Event Scale-Revised (IES-R) assesses posttraumatic symptom severity [42]. It consists of 3 subscales: intrusions, avoidance, and hyperarousal. It has 22 items rated on a 0 to 5 Likert scale (subscale scores: 0-35 for intrusions and hyperarousal and 0-40 for avoidance; Cronbach α=0.90 for intrusions, α=0.80 for avoidance, and α=0.90 for hyperarousal).

The German short version of the Pregnancy-Related Anxiety Questionnaire-Revised (PRAQ-R) was used to measure pregnancy-specific anxiety [43]. It consists of 10 items, rated 1 to 5 points (total 10-50; higher score = more anxiety; Cronbach α=0.82).

The Social Support Questionnaire (F-SozU) K14 is a 14-item short version assessing perceived/anticipated social support [44]. It uses a 5-point Likert scale (Cronbach α=0.94).

The World Health Organization Quality of Life-BREF (WHOQOL-BREF) consists of 26 items assessing quality of life across 4 domains (physical, psychological, social, and environmental) [45]. Scores range from 0 to 100 (Cronbach α≥0.75).

The Partnership Questionnaire (PFB) is a German 30-item questionnaire measuring partnership quality [46]. It consists of 3 subscales (10 items each): conflict, tenderness, and communication. It uses a 0 to 3 frequency scale (total 0-90; Cronbach α=0.88-0.93).

The German 21-item version of the MAUQ evaluates the usability of mobile health (mHealth) apps [47]. It consists of 3 subscales: ease of use, interface satisfaction, and usefulness, rated on a 7-point Likert scale (range 1-7; higher mean=better usability).

The SUS is a 10-item established usability tool, validated in German [48]. It was completed before the interview to reduce response bias. It uses a 5-point Likert scale, scored 0 to 100 (average=68 and best=100).

For this study, we performed descriptive analyses of sociodemographic and patient history data. Results from the questionnaires were reported descriptively. We used 2-tailed paired *t* tests to evaluate changes in knowledge and use of pregnancy-related services. We then correlated data from our psychometric questionnaires with changes in knowledge and use using the Pearson correlation coefficient. Participants were excluded if they missed more than 10% of items on the questionnaire. Missing values were replaced by the mean scale value. We set the level of significance at *P*<.05. Statistical tests were performed using SPSS (version 30; IBM Corp) and graphed with GraphPad (version 10; GraphPad Software).

### Qualitative Data Collection and Analysis

Qualitative data collection was conducted after a 2-week test phase with 13 outpatients and 17 inpatients, using semistructured interviews. The interview guide was reviewed and revised by the study team throughout the process (Multimedia Appendix 3). The interview covered topics related to usability, design, content (particularly questionnaires, individual care plans, advice chapters, videos, and networking with HSPs), as well as participants’ overall opinions and experiences.

The interviews were audio-recorded after participants had given their written consent and transcribed verbatim. Field notes were taken during each interview to capture contextual information and cross-check with the transcriptions. Each interview lasted between 12 and 43 minutes, with a median duration of 23 minutes 20 seconds.

We conducted a thematic analysis at the manifest content level using an inductive approach. After familiarizing themselves with the data, 2 independent researchers separately reviewed and line-by-line coded the transcripts, assigning descriptive labels to meaningful segments of text. The initial codes were then compared, discussed, and refined through iterative team discussions with our principal investigator, and related codes were grouped into themes and subthemes until consensus was reached. Representative quotes illustrating each theme were selected and translated into English for reporting [37,49]. Data saturation was reached after conducting 30 semistructured interviews, when no new themes or insights emerged across 3 consecutive interviews, as confirmed through iterative coding using MAXQDA qualitative data analysis software (MAXQDA 2022, release 22.7.0).

## Results

### Qualitative Findings

#### Overview

A total of 53 women agreed to participate in the pilot study. Of these, 30 agreed to be interviewed at the end of the test phase, and 35 completed the SUS after their test phase. The remaining participants dropped out over the course of the study. Known reasons for dropping out were other issues concerning their respective pregnancy, technical errors, or lack of time (Figure 3).

Reasons for nonparticipation included lack of mental capacity (n=7), lack of time (n=6), lack of interest in the app (n=5), already using a pregnancy app (n=3), insufficient storage space (n=1), and distant place of residence (n=1).

The median maternal age was 33 (range 21-41; IQR 6) years, and the median gestational age was 30 (range 20-34; IQR 6) weeks. After analyzing the data, 2 main categories, “usability” and “features,” along with 7 subcategories, were identified. These categories were further organized into limiting factors and success factors based on the codes derived from the data.

**Figure 3:**
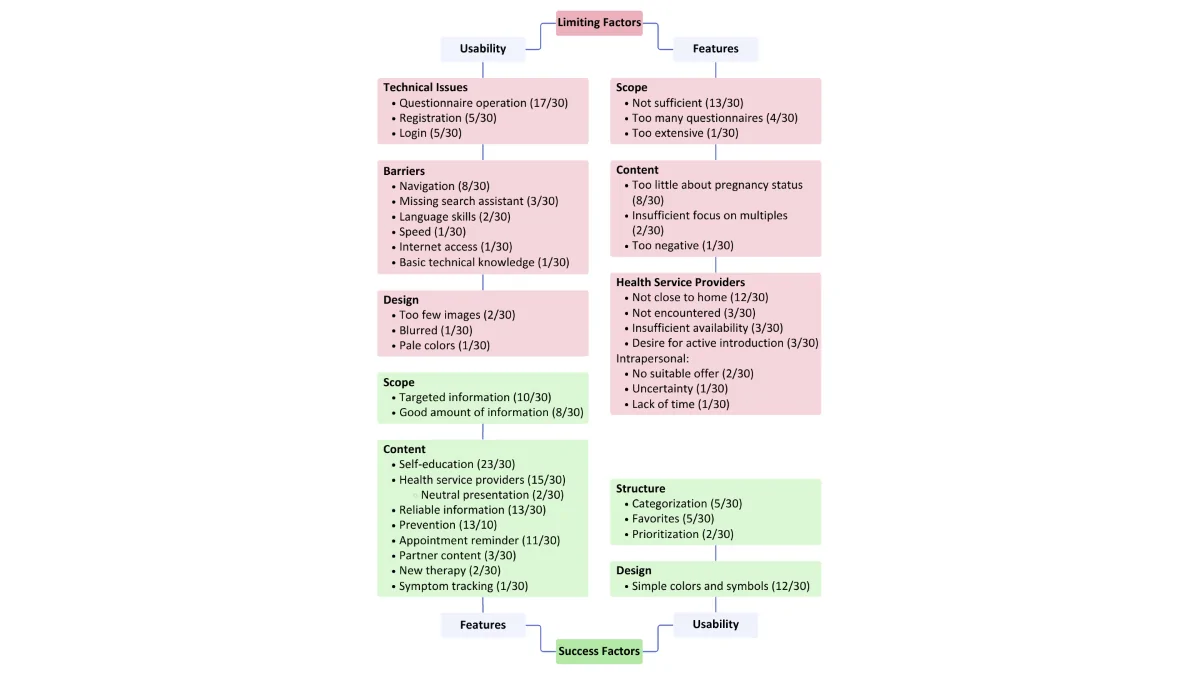
Findings on success and limiting factors from 30 semistructured interviews.

#### Limiting Factors

##### Usability

During the pilot study, participants identified several technical errors, particularly when filling out the questionnaires or registering in the app. These issues were perceived as disruptive to the user experience, though they were continuously addressed and corrected technically.

Some participants reported difficulties with navigation (n=8), noting that it was initially unclear where to find specific content. Opinions were divided on whether an explanatory guide at the start of the app would have been helpful. Some participants desired such a guide, while others considered it unnecessary or annoying.

Other barriers identified included the need for good language skills (n=2), basic technical knowledge (n=1), and internet access (n=1).

My mother is not very good at German and also not tech-savvy. For her it would possibly be a complete disaster. She wouldn’t be able to handle it. It always depends on the person’s (...) knowledge regarding language and technology.

Participant 2

##### App Contents

Participants frequently noted that the app did not fully meet their information needs (n=13), and many expressed a desire for more information on specific topics. Some felt that the level of detail was insufficient, especially on subjects that were particularly relevant or urgent to them at the time.

I would have thought that in some extent there would have been a bit more information. So just that you can go even further in details, because I found some things a bit brief.

Participant 7

A common request was for more information about ongoing processes in the current week of pregnancy and child development (n=8).

I was hoping for a bit more information, for example about the week of pregnancy or something about the changes in the mother’s body. I thought it would be nice if there was a bit more information of this kind. For example, that the belly gets hard from time to time or that training contractions can occur and things like that.

Participant 12

Additionally, the 2 participants expecting twins expressed a need for more extensive personalization of the app content for women expecting multiples (n=2).

So when you open the app, it says (...) your child is this big and then I always think to myself, NO, my CHILDREN. (...) (I would wish) for a bit of additional information, especially if you use the app and have twins. I then saw that there was an extra category with twins, where there were also some articles (...) and sometimes I think it would be nice to go into it again briefly if there is something specific about the topic.

Participant 24

However, there were individual participants who found the abundance of information overwhelming (n=1), expressed dissatisfaction with the number of questionnaires (n=4), or felt that some articles, particularly those focusing on diseases, conveyed a negative tone (n=1).

I think my biggest problem actually is, it is too much. That you have too many questionnaires, too much text, too much/ I think that was what made me so, yeah, uh, repulsed, that no matter what I looked at, I had a huge chunk of text first.

Participant 26

##### HSPs

Regarding the integration and targeted referral to HSPs, participants emphasized the need for services close to their homes (n=13). In some cases, this was specifically cited as a reason why contact with HSPs was not established. Conversely, some participants noted that proximity could facilitate contact with service providers.

So personally, it didn’t help me that much, because they are not near our location.

Participant 15

Some participants were unaware of the specific section for HSPs in the app (n=3), which may be related to navigation challenges. Others expressed a desire for lower-barrier contact options, such as a contact form or email addresses (n=3). Several participants requested more proactive encouragement to engage with HSPs (n=3), such as pop-up messages or positive reinforcement encouraging users to access available support services.

Somehow say in one sentence (...) that it is alright if you need help, and everyone needs help sometimes and that you can contact them (HSPs) anytime and don’t need to be afraid.

Participant 5

Intrapersonal barriers to contacting HSPs included unsuitable providers (n=2), lack of time (n=1), and uncertainty (n=1). Most study participants indicated that they did not perceive a current need for HSPs (n=14) or felt their needs were already adequately addressed (n=2).

The first birth was kind of traumatic for me and I would have liked to have had a conversation, a consultation beforehand, because I’m quite scared, very scared, of the second one. I would have liked to have made an appointment somehow, but I didn’t due to time constraints. But that’s what the app (...) was actually good for, just to have a look where that is possible.

Participant 23

##### Personalization

The piloted version of the app was occasionally criticized for not being personalized enough (n=12). Dates for upcoming appointments did not match for some pregnant women, for example (n=4). Others mentioned receiving suggestions that were not relevant to them, especially in the information articles (n=7). In this context, one can differentiate between objectively inappropriate content, such as suggesting sports exercises during medically prescribed bed rest (n=2), and subjectively inappropriate content, such as recommending psychological support services (n=5).

Sometimes I had the feeling that at least at the beginning it wasn’t tailored to me or didn’t match what I had entered in the questionnaires, or sometimes I think things were simply not polled in the questionnaires, so that some unfitting content was shown.

Participant 29

#### Success Factors

##### Usability

The breakdown of topics into headings and subtopics was perceived as clear and allowed users to filter content according to their own interests (n=5). Additionally, the ability to save favorites (n=5) and the prioritization of important information within articles (n=2) were seen as contributing factors to the app’s usability.

So I thought, this is my chance, because the doctors have explained it to me and now I’m curious to see how the app explains it to me and I thought it was really great how the app has structured and subdivided it (the self-educative contents) and that you always have a kind of post-it where you can find keyword phrases, the most important ones. (...) I thought that was really good.

Participant 20

##### Graphic Design

The positive impact of the graphic design was highlighted multiple times. The color scheme and overall presentation were reported to create a positive, pleasant, and appealing atmosphere (n=12).

That’s really great. I find it really calming. It’s not extremely bright or anything, so I found it really relaxing.

Participant 27

##### Content

Participants frequently mentioned that the amount of content (n=8) and the concise, focused information (n=10) were well received.

I think it was always broken down (...) to what is really relevant.

Participant 14

Most study participants emphasized that the self-educational content was highly beneficial (n=23).

I really read through a lot of things and they were really very informative. You really had this feeling afterwards, that you haven’t just scratched the surface a little, but you’ve really generated knowledge about a topic.

Participant 28

Additionally, participants noted that the content, being authored by medical professionals, conveyed reliable and trustworthy information (n=13).

That’s exactly what was always discussed with the doctors. So I concluded, great, (...) you can really rely on it.

Participant 14

Study participants also felt that the app contributed to prevention (n=14). Topics particularly mentioned included sports during pregnancy (n=7), psychological topics (n=4), and medical topics such as vaccinations (n=1), blood pressure (n=1), and wound healing (n=1).

The integrated appointment bar, which provided an overview of upcoming treatments and preventive care appointments, was considered helpful (n=11). It also served as a reminder for some participants about upcoming appointments (n=3).

For example, pertussis vaccination was something I’d heard of before, that you can get it again. But I think I’d forgotten it with all the things going on. That’s why it was such a good reminder. I immediately made plans to discuss this with the gynecologist again.

Participant 5

Two participants also discovered new or alternative treatment options via the app, which they pursued after consulting with their treatment teams.

It also said that you can freeze the colostrum after massage and so I asked the nurse today and she said: “Yes, of course, we can do that.” I started right away today (...) And this thanks to the app. I was really happy about it.

Participant 20

The integration of partners and different family constellations into the questionnaires and app content was positively received, fostering conversations and reflection (n=3).

It was then somehow about the spouse. I really liked this questionnaire because it made you aware of all the things your partner actually does for you and how positive this relationship is.

Participant 5

The integration of HSPs was also seen as valuable by the study participants (n=15). The information and contact options provided were considered informative (n=15), and the neutrality of the information was highlighted as a positive aspect (n=2).

Half of the study participants reported that they had discovered new HSPs, and 3 participants even contacted HSPs during the trial phase.

But then I realized once again how many offers there really are in Germany for young parents, for siblings, for partnerships in general.

Participant 20

##### Personalization

Many respondents indicated that the personalized approach in individual care plans was accurate (n=18). They found the pertinent information well communicated, which enhanced their engagement. Moreover, it was noted that this personalized approach fostered a stronger sense of individual connection and reduced the time required for gathering information.

With the questionnaires you must take ten minutes of your time, but you benefit from it because to a certain extent it gets tailored to you and then you’re not so overloaded with information.

Participant 20

##### Feelings

Some pregnant women perceived a positive change in their own feelings (n=7). These changes were described as a feeling of being more informed (n=5) and more confident (n=2).

I trust the doctors and midwives who have written this. And this gives me a bit more security.

Participant 13

Of the 30 pregnant women interviewed, 29 indicated that they saw a need for an app of this type. Likewise, 29 interviewees stated they would recommend the app to other pregnant women.

### Quantitative Findings

#### Sociodemographic Measures

Of the 53 pregnant women who were willing to participate, 35 actively used the app and filled out questionnaires. Due to technological errors, demographic data and risk factors were collected from 35 participants. The sample size differs between analyses due to missing values. Our cohort shows typical features of the population in Heidelberg, with a high number of academics, low migration background, and an average net household income comparable to Germany’s median net household income [38]. Most participants are on pregnancy leave and nulliparous with a singleton pregnancy. We did not have single mothers in our cohort: two-thirds of our participants are married, and one-third live in a relationship. Around 30% have a history of mental health disorders, and more than half of the patients expressed some sort of psychosocial burden (eg, financial problems or partnership distress). More than half of the patients self-reported 1 or more pregnancy complications, and almost half of them demonstrated preexisting conditions (Table 1).

**Table 1:** Demographic data of the study participants from the in-app data collection.

Variable and category		Frequency, n (%)
**Age (n=35)**		
	<20	0 (0)
	20–35	24 (68.6)
	>35	11 (31.4)
	Median (IQR)	33 (6)
**Gravidity (n=35)**		
	G1	19 (54.3)
	G2	7 (20)
	G3	5 (14.3)
	G4	4 (11.4)
**Parity (n=35)**		
	P0	24 (68.6)
	P1	8 (22.9)
	P2	2 (5.7)
	P3	1 (2.9)
**Number of fetuses (n=31)**		
	Singleton pregnancy	25 (80.6)
	Multiple pregnancy	6 (19.4)
**Psychosocial factors (n=31)**		
	History of mental disorder	9 (29)
	Psychosocial burden	16 (51.6)
**Self-reported pregnancy complications (n=31)**		
	Cervical insufficiency	7 (22.6)
	Heavy nausea and vomiting	4 (12.9)
	Fetal growth restriction	3 (9.7)
	Premature contractions	2 (6.5)
	Infection	2 (6.5)
	Coagulation disorder	2 (6.5)
	Gestational diabetes	1 (3.2)
	Hypertension	1 (3.2)
	Premature rupture of membranes	1 (3.2)
	None	14 (45.2)
**Preexisting diseases (n=31)**		
	Thyroid disease	5 (14.3)
	Coagulation disorder or thrombosis	3 (9.7)
	Gastrointestinal disease	2 (6.5)
	Asthma	2 (6.5)
	Hypertension	1 (3.2)
	Neurological disorders	1 (3.2)
	Kidney disease	1 (3.2)
	Autoimmune disease	1 (3.2)
	Endometriosis	1 (3.2)
	Uterine fibroids	1 (3.2)
	Premature ovarian insufficiency	1 (3.2)
	None	17 (54.8)
**In-app risk grouping (n=31)**		
	Psychological	17 (54.8)
	Medical	8 (25.8)
	Prematurity	16 (51.6)
	Psychosocial	6 (19.4)
**Relationship status (n=31)**		
	Married	19 (61.3)
	Relationship	11 (35.5)
	Single	1 (3.2)
**Migration background (n=30)**		
	Yes	2 (6.5)
	No	28 (93.3)
**Higher education (n=31)**		
	Yes	14 (45.2)
	No	17 (54.8)
**Monthly net household income (€^a^; n=31)**		
	<1500	2 (6.5)
	1500–2999	19 (61.3)
	3000–4999	7 (22.6)
	5000–8000	3 (9.7)

^a^EUR €1=US $0.99 as of September 22, 2022.

#### Psychometric Measures

The psychometric test results presented aim to offer a comprehensive overview of our participants’ state of mind while at the same time testing the feasibility of extensive measurements through the app (Table 2). We analyzed all questionnaires descriptively. Participants showed elevated psychosocial burden: median EPDS was 8 (IQR 4-12), indicating mild depressive symptoms, with 43.5% (10/23) scoring >9 (at least mild depression). State anxiety (STAI-S) median was 47 (IQR 35.5-52), with 72% (18/25) exceeding the state anxiety cutoff (≥40); trait anxiety (STAI-T) median was 37.5 (IQR 30.25-48), with 50% (12/24) above cutoff (≥40), indicating moderate-to-high anxiety and moderate anxiety predisposition. Pregnancy-related anxiety was moderate (median 25.5, IQR 18-30; n=22). PSS was high (median 27, IQR 22-33; n=25). IES-R scores showed a wide range (intrusion: median 13, IQR 6-25; avoidance: median 9, IQR 2-18; hyperarousal: median 10, IQR 3-16; n=15), but no PTSD diagnosis [39]. Maternal-fetal attachment was strong (median 133.5, IQR 116-143.5; n=26). Quality of life was moderate (median 14, IQR 14-16; n=19). Partnership flexibility (PFB) was relatively high (median 69, IQR 63.25-80; n=22), and social support was high (median 64, IQR 57-67; n=23).

**Table 2:** Psychometric measures, in-app data collection 1 and 2.

Psychometric instrument (n, score range)	Value, mean (SD)
EPDS^a^ (n=23, 0-30)	8.35 (4.80)
F-SozU^b^ K14 (n=23, 1-5)	4.34 (0.58)
IES^c^-intrusion (n=15, 0-35)	14.33 (10.44)
IES-avoidance (n=15, 0-40)	9.87 (8.03)
IES-hyperarousal (n=15, 0-35)	10.4 (7.47)
MFAS^d^ (n=26, 24-168)	130.31 (17.12)
PFB^e^ (n=22, 0-90)	69.27 (10.99)
PRAQ-R^f^ (n=22, 10-50)	25.36 (7.57)
PSS^g^ (n=25, 0-40)	17.12 (6.65)
STAI-S^h^ (n=25, 20-80)	45.56 (11.84)
STAI-T^i^ (n=24, 20-80)	37.75 (10.40)
WHOQOL-BREF^j^ physical health (n=19, 0-100)	59.59 (18.71)
WHOQOL-BREF psychological health (n=19, 0-100)	71.31 (11.05)
WHOQOL-BREF social relationships (n=19, 0-100)	70.61 (15.05)
WHOQOL-BREF environment (n=19, 0-100)	75.99 (8.66)

^a^EPDS: Edinburgh Postnatal Depression Scale.

^b^F-SozU: Social Support Questionnaire.

^c^IES-R: Impact of Event Scale.

^d^MFAS: Maternal Fetal Attachment Scale.

^e^PFB: Partnership Questionnaire.

^f^PRAQ-R: Pregnancy-Related Anxiety Questionnaire-Revised.

^g^PSS: Perceived Stress Scale.

^h^STAI-S: State-Trait Anxiety Inventory-state scale.

^i^STAI-T: State-Trait Anxiety Inventory-trait scale.

^j^WHOQOL-BREF: World Health Organization Quality of Life-BREF.

#### Usability

Like our qualitative results, we found that users were content with the app’s usability. Our in-app MAUQ was answered by 20 out of 35 participants. The SUS was therefore additionally tested with 35 participants before our semistructured interviews. The median score for system usability was 80 (IQR 17.5) and lies within 90th percentile. The median score for the MAUQ was 111 (IQR 28), indicating excellent usability.

#### Knowledge and Use of HSPs

When asked about their knowledge and use of HSPs, we found that already after this 2-week trial, knowledge of many services increased significantly in 31 participants, whereas use only increased in 2 cases, namely nutrition counseling and hospitals (Table 3). Knowledge of relationship counseling increased significantly with trait and state anxiety (STAI-T: *P*=.02; STAI-S: *P*=.02) as well as with perceived stress (PSS: *P*=.03). With an increasing sense of self-efficacy, use of relationship counseling (*P*=.04), psychotherapy (*P*=.04), and postnatal recovery courses (*P*=.047) significantly increased. A significant increase in knowledge of nutrition counseling with state anxiety was observed (*P*=.03).

**Table 3:** Knowledge and use of health service providers, in-app data collection of 31 study participants.

Health service providers	Knowledge (T1-T2), *P* value	Use (T1-T2), *P* value
Pregnancy counseling	.01	.59
Addiction counseling	.03	.60
Consultation for parents with infants	.72	.88
Breastfeeding counseling	.19	.43
Counseling for regulatory disorders	.34	.39
Telemedical counseling	.045	.57
Counseling for single mothers	.33	.88
Relationship counseling	.66	.84
Nutrition counseling	.045	.03
Guidance services	.10	>.99
Midwife services	.37	.33
Family midwives	.17	.33
Self-help groups	.02	.88
Volunteer family mentors	.05	.83
Parent associations	.04	.52
Psychological counseling for pregnancy and motherhood	.13	.88
Relief services	.02	.54
Social services	.02	.21
Frühe Hilfen (early help)	.10	.39
Early intervention center	.26	.65
Clinics, hospitals	.18	.003
Community-based doctors	.05	.26
Social pediatric centers	.06	.53
Physiotherapy	.13	.32
Psychotherapy	.36	.75
Mother-child units	.14	.42
Prenatal classes	.37	.24
Parenting courses	.59	.87
Parent-child groups	.12	.21
Postnatal recovery courses	.08	.23
Sibling preparation classes	.12	.52
Parent counseling, family, or educational counseling	.12	.41

### Integration of Quantitative and Qualitative Findings

In this convergent mixed methods design, quantitative and qualitative results were analyzed separately and then integrated to provide a more comprehensive understanding of the app’s usability and the feasibility of the planned RCT tested in a high-risk patient cohort.

The high usability scores (SUS and MAUQ) converged with interview reports describing clear structure, appealing design, and helpful self-educational content, indicating a consistently positive user experience across methods. However, qualitative data revealed important limitations not captured by global scores: technical errors during questionnaire completion and registration resulted in dropouts, as did navigation difficulties and barriers for users with limited German language skills or low technical literacy. This triangulation indicates that while the app is highly usable for educated, digitally literate users, it may present challenges for more vulnerable populations.

Relatively high anxiety scores within the group at the beginning coincided with patients reporting a clear need for a navigator and a feeling of greater security through self-education. Concerning feasibility, interviews showed high demand and a clear recommendation for other users. On the other hand, psychometric tests showed an extensive dropout rate of up to 50%, which is explained by the interviews indicating discontent with overwhelming amounts of information and questionnaires among some participants. Both data sources confirm the app’s success in increasing HSP knowledge (quantitative: significant increase in 31 participants; qualitative: half discovered new HSPs, n=15 found information valuable). Quantitative analyses showed that knowledge of relationship counseling increased with anxiety, and self-efficacy predicted use of relationship counseling, psychotherapy, and postnatal recovery courses, supporting qualitative requests for proactive encouragement. Nevertheless, qualitative barriers (distance and unawareness of HSP section due to navigation issues, n=3) explain the low use despite knowledge gains.

## Discussion

### Principal Findings

Our study aimed to evaluate the feasibility and usability of our digital navigator by exploring barriers and success factors through a mixed methods approach. Our results demonstrated that implementation of our mobile navigator is feasible and has sufficiently high usability. However, it also revealed interindividual variations in the needs and expectations of women with high-risk pregnancies regarding these applications. It is mandatory to incorporate the needs and consider the barriers to the application in our future research project and to adjust it adequately. The digital navigator is intended to increase the attractiveness and clarity of the diverse care services available for families experiencing psychosocial burden and to improve care coordination in essential, nonphysician care settings. This is only possible with high usability for the individual. Future research will determine whether use of the digital navigator leads to an increase in knowledge about and use of the services presented through the navigator. Targeted, easy-to-understand information transfer through links to established evidence-based information modules and existing counseling services could strengthen parental competence. Increased use could contribute to optimization of medical outcomes.

### Content and Usability

Our qualitative findings highlight the importance of both trust in medical content and individualized in-depth information. We identified interindividual differences in information needs: while 13 participants desired more detailed information and 10 felt certain topics were not covered in sufficient depth, 6 found some content overwhelming. Personalization, particularly in information depth, may represent a significant improvement for eHealth apps. Although Conway et al [40] argued that there is not enough evidence to confirm the benefits of tailored information in eHealth, our study population showed a striking desire for tailored content—consistent with findings from other pilot studies on pregnancy-specific mHealth tools [41,42]. Since most pregnant women obtain information online, which strongly influences their decision-making, reliable sources are crucial [43]. Our digital navigator was effective due to its focused, self-educational content and the perceived trustworthiness of the information, which participants associated with the authorship by HSPs.

High usability scores and significant increases in knowledge of support services suggest that digital tools may be able to bridge informational gaps in perinatal care—particularly for vulnerable populations. Language barriers, lack of internet access, and limited technical knowledge emerged as challenges to successful use. Although the usability of our navigator was found to be sufficient for all participants, future development should prioritize accessibility, including offering translations in native languages [44].

### mHealth in Perinatal Care

mHealth services have already been shown to positively influence perinatal care [27]. While our study design does not allow conclusions about improved outcomes, it suggests that mHealth tools can increase users’ focus on their health, potentially enhancing self-efficacy. Importantly, participants expressed that the app supported their sense of control and emotional well-being—factors known to influence maternal health outcomes. This is also reflected in our results, showing that with an increasing sense of self-efficacy, use of relationship counseling, psychotherapy, and postnatal recovery courses increased significantly.

mHealth apps, including our navigator, have the potential to address deficits in information provision regarding pregnancy, birth, and the postnatal period. However, both high-quality content and integration into health care systems are essential so that pregnant women can meet their information needs and be optimally supported in their decision-making processes [43,45]. Our results align with other studies showing that reliable information is desirable for pregnant women, that information gain through digital applications is perceived as positive, and that overviews of appointments and therapies are perceived as helpful [46,47].

### Integration of HSPs

There are various prerequisites for integrating HSPs into apps, such as ensuring neutrality, active introduction, and proximity to users’ homes. The latter was confirmed by Ney et al [50], who were also able to identify low costs as a variable for higher use. Studies have shown that eHealth services, as well as one’s own internet research about suitable offers, can increase the use of prenatal consultations or health literacy programs [48,50]. In our short study interval, 3 participants contacted integrated HSPs by using the app, which indicates that the use of HSPs could be increased by our navigator.

Data on the use of HSPs for young families from the Federal Initiative for Early Prevention in Germany already suggested that there might be a connection between the use of HSPs and the need to organize this support on one’s own initiative [30]. This aligns with our participants’ expressed need for active introductions to available services through the app.

While knowledge acquisition improved in our study group, actual use of support services increased only marginally. This discrepancy suggests that informational access alone may not be sufficient to drive behavioral changes. Here we saw an increase especially for clinics and nutrition counseling, which could be biased due to the inpatient portion of the study group. Future versions of the app should therefore incorporate behavioral nudges, appointment reminders, or integrated referral features to improve uptake.

### Limitations

A limitation of our study is the sample composition. Participants treated at the University Women’s Hospital Heidelberg may not represent the broader population in Germany, particularly in terms of academic background and especially because most of them belong to a high-risk population since they are hospitalized. The findings may therefore not be applicable to the general population. In more diverse settings—which will be evaluated within the multicenter RCT—usability might be lower due to factors like digital access barriers, language challenges, or lower health literacy.

We achieved information saturation on barriers and success factors after 30 interviews, which is considered appropriate for a qualitative study [51]. The semistructured interviews provided broad and deep insight into the users’ experience. Our results were additionally restrained due to the very short test phase and small sample size. Due to hospitalization and time constraints, no measurable effects regarding higher use of HSPs could be seen. Long-term effects on psychometric measures were not researched and therefore cannot be correlated with the short-term high usability scores. The rather small sample size resulted in some statistically significant findings, which may rather reflect chance than meaningful effects. The high dropout rate for psychometric testing might indicate low feasibility and therefore future problems in reaching our aspired research goals. By using the SUS and MAUQ, we confirmed a sufficiently high usability score, which was supported by the interview findings. This alignment between qualitative and quantitative data reinforces the reliability of our results.

### Conclusion

The results of our pilot study indicate that pregnant women can benefit from our digital navigator. The usability was rated highly, and a need for apps of this kind was expressed. The identified barriers will be considered in the further development of Family eNav and other digital care programs.

Our follow-up multicenter RCT must explore whether the use of our digital navigator can lead to increased use of help and support services.

## Appendix

Multimedia Appendix 1 GRAMMS checklist.

Multimedia Appendix 2 Questionnaire Health Service Providers, translated.

Multimedia Appendix 3 A Interview guide, translated. B System Usability Scale, translated.

## Abbreviations

EPDS: Edinburgh Postnatal Depression Scale
F-SozU: Social Support Questionnaire
GRAMMS: Good Reporting of a Mixed Methods Study
HSP: health service provider
IES-R: Impact of Event Scale-Revised
MAUQ: mHealth App Usability Questionnaire
MFAS: Maternal Fetal Attachment Scale
mHealth: mobile health
PFB: Partnership Questionnaire
PRAQ-R: Pregnancy-Related Anxiety Questionnaire-Revised
PSS: Perceived Stress Scale
Q-HSP: Questionnaire on Knowledge and Utilization of Health Service Providers
RCT: randomized controlled trial
STAI: State-Trait Anxiety Inventory
STAI-S: STAI state scale
STAI-T: STAI trait scale
SUS: System Usability Scale
WHOQOL-BREF: World Health Organization Quality of Life-BREF

## Data Availability

The data sets generated and analyzed during this study are not publicly available due to participant privacy restrictions but are available from the corresponding author upon reasonable request. Deidentified quantitative data and interview excerpts may be shared in accordance with institutional ethical guidelines of the University Women’s Hospital Heidelberg.
