# Supplementary material for: Feasibility and Usability of a Digital Perinatal Navigator for High-Risk Pregnancies: Mixed Methods Study
Source: J Med Internet Res. 2026 Oct 8;28:e88015. doi: 10.2196/88015 (PMC13691458; doi:10.2196/88015)
Supplement: Supplementary file 1 [file jmir_v28i1e88015_app1.pdf]

Good Reporting of A Mixed Methods Study (GRAMMS) checklist Guideline

1. Describe the justification for using a mixed methods approach to the research question

Section: Methods under Participants and Study Design pg. 9

2. Describe the design in terms of the purpose, priority and sequence of methods

Section: Methods under Participants and Study Design as well as Data Collection and Analysis pg. 9-10

3. Describe each method in terms of sampling, data collection and analysis Procedures

Section: Methods under Data Collection and Analysis pg. 10-13

4. Describe where integration has occurred, how it has occurred and who has participated in it

Section: Methods under Participants and Study Design pg. 9 & Results under Integration of quantitative and qualitative findings pg. 25

5. Describe any limitation of one method associated with the present of the other method

Section: Discussion under Limitations pg. 28

6. Describe any insights gained from mixing or integrating methods

Section: Discussion pg. 25-28

Literature:

O'Cathain A, Murphy E, Nicholl J. The quality of mixed methods studies in health services research. J Health Serv Res Policy. 2008;13: 92-98.
