# Supplementary material for: Feasibility and Usability of a Digital Perinatal Navigator for High-Risk Pregnancies: Mixed Methods Study
Source: J Med Internet Res. 2026 Oct 8;28:e88015. doi: 10.2196/88015 (PMC13691458; doi:10.2196/88015)
Supplement: Supplementary file 2 [file jmir_v28i1e88015_app2.docx]

Survey „Knowledge and Utilization of Psychosocial Services “

# Knowledge

Scale 0 to 4

0 – no knowledge

1 – little knowledge

2 – moderat knowledge

3 – good knowledge

4 – extensive knowledge

| Services | no knowledge | little knowledge | moderat knowledge | good knowledge | extensive knowledge |
| --- | --- | --- | --- | --- | --- |
| Counseling Services |  | | | | |
| Pregnancy counseling (e.g., Pro Familia) | O | O | O | O | O |
| Addiction counseling | O | O | O | O | O |
| Psychological counseling for pregnant women and mothers | O | O | O | O | O |
| Outpatient clinic for parents of infants/young children | O | O | O | O | O |
| Parent counseling, family or parenting counseling | O | O | O | O | O |
| Breastfeeding counseling | O | O | O | O | O |
| Counseling for regulation disorders (crying/sleeping/feeding) | O | O | O | O | O |
| Telephone or online counseling (parent hotline, Nummer gegen Kummer) | O | O | O | O | O |
| Counseling for single parents | O | O | O | O | O |
| Relationship counseling | O | O | O | O | O |
| Nutrition counseling | O | O | O | O | O |
| Navigation services (baby navigators, family navigators) | O | O | O | O | O |
| Midwifery |  | | | | |
| Midwife assistance | O | O | O | O | O |
| Family midwives | O | O | O | O | O |
| Lay services / volunteer work |  | | | | |
| Self-help groups | O | O | O | O | O |
| Volunteer sponsors, family sponsorships | O | O | O | O | O |
| Parent associations | O | O | O | O | O |
| Pastoral care | O | O | O | O | O |
| Overarching institutions |  | | | | |
| Youth welfare office, general social services | O | O | O | O | O |
| Frühe Hilfen | O | O | O | O | O |
| Early intervention centers | O | O | O | O | O |
| Medical services |  | | | | |
| Clinics, hospitals | O | O | O | O | O |
| Private practices | O | O | O | O | O |
| Social-pediatric centers | O | O | O | O | O |
| Physiotherapy | O | O | O | O | O |
| Psychological services |  |  |  |  |  |
| Psychotherapy | O | O | O | O | O |
| Mother-child units | O | O | O | O | O |
| Courses |  |  |  |  |  |
| Birth preparation classes | O | O | O | O | O |
| Parent courses/parent training | O | O | O | O | O |
| Parent-child groups (breastfeeding, crawling groups, baby swimming, PEKiP) | O | O | O | O | O |
| Postpartum recovery courses | O | O | O | O | O |
| Sibling preparation courses | O | O | O | O | O |

# Utilization

Scale 0 to 4

0 – not utilized

1 – little utilized

2 – moderately utilized

3 – well utilized

4 – highly utilized

| Services | not utilized | little utilized | moderately utilized | well utilized | highly utilized |
| --- | --- | --- | --- | --- | --- |
| Counseling Services |  | | | | |
| Pregnancy counseling (e.g., Pro Familia) | O | O | O | O | O |
| Addiction counseling | O | O | O | O | O |
| Psychological counseling for pregnant women and mothers | O | O | O | O | O |
| Outpatient clinic for parents of infants/young children | O | O | O | O | O |
| Parent counseling, family or parenting counseling | O | O | O | O | O |
| Breastfeeding counseling | O | O | O | O | O |
| Counseling for regulation disorders (crying/sleeping/feeding) | O | O | O | O | O |
| Telephone or online counseling (parent hotline, Nummer gegen Kummer) | O | O | O | O | O |
| Counseling for single parents | O | O | O | O | O |
| Relationship counseling | O | O | O | O | O |
| Nutrition counseling | O | O | O | O | O |
| Navigation services (baby navigators, family navigators) | O | O | O | O | O |
| Midwifery |  | | | | |
| Midwife assistance | O | O | O | O | O |
| Family midwives | O | O | O | O | O |
| Lay services / volunteer work |  | | | | |
| Self-help groups | O | O | O | O | O |
| Volunteer sponsors, family sponsorships | O | O | O | O | O |
| Parent associations | O | O | O | O | O |
| Pastoral care | O | O | O | O | O |
| Overarching institutions |  | | | | |
| Youth welfare office, general social services | O | O | O | O | O |
| Frühe Hilfen | O | O | O | O | O |
| Early intervention centers | O | O | O | O | O |
| Medical services |  | | | | |
| Clinics, hospitals | O | O | O | O | O |
| Private practices | O | O | O | O | O |
| Social-pediatric centers | O | O | O | O | O |
| Physiotherapy | O | O | O | O | O |
| Psychological services |  |  |  |  |  |
| Psychotherapy | O | O | O | O | O |
| Mother-child units | O | O | O | O | O |
| Courses |  |  |  |  |  |
| Birth preparation classes | O | O | O | O | O |
| Parent courses/parent training | O | O | O | O | O |
| Parent-child groups (breastfeeding, crawling groups, baby swimming, PEKiP) | O | O | O | O | O |
| Postpartum recovery courses | O | O | O | O | O |
| Sibling preparation courses | O | O | O | O | O |
