# Supplementary material for: Feasibility and Usability of a Digital Perinatal Navigator for High-Risk Pregnancies: Mixed Methods Study
Source: J Med Internet Res. 2026 Oct 8;28:e88015. doi: 10.2196/88015 (PMC13691458; doi:10.2196/88015)
Supplement: Supplementary file 3 [file jmir_v28i1e88015_app3.docx]

# Anhang 1: Interviewleitfaden

Im Interview wurden Schwerpunkte auf die folgenden Themen gelegt: Handhabung, Darstellung, Inhalte, Einschätzung der App (Motivation, Empfehlung, Mehrwert)

Fett markierte Unterpunkte fungierten dabei als Leitfrage und wurden im Verlauf durch andere Fragen tiefergehend exploriert. Bei Bedarf wurden Fragen im Interview angepasst oder weitere Themen ergänzt.

1. Einführung vor Beginn des Interviews

2. Handhabung, wenn schlecht oder nicht gut: Warum nicht?

2.1. Wie sind Sie grundsätzlich mit der MamaHerz-App zurechtgekommen? Gab es

Schwierigkeiten bei der allgemeinen Handhabung?

2.2. Was könnte Ihrer Meinung nach die Nutzung der App erleichtern?

2.3. Sind Sie auf technische Probleme gestoßen?

2.4. Wie kamen Sie mit der Navigation zurecht? *(Scrollen und Antippen)?*

2.5. Haben Sie die Inhalte, die Sie interessieren, schnell und einfach gefunden? Wie

übersichtlich fanden Sie die Darstellung der Inhalte?

2.6. Wünschen Sie sich mehr Informationen oder Instruktionen zur Nutzung der App?

3. Darstellung

3.1. Wie hat Ihnen die graphischen Darstellung des Portals gefallen?

3.2. … Was würden Sie gerne ändern? / Was hat Ihnen besonders gut gefallen?

3.3. Wie empfanden Sie die verwendete Schrift- und ICON-Größe?

3.4. Wie fanden Sie die Ansprache per “Du”? Wären Sie lieber mit “Sie” angesprochen

worden?

4. Inhalte

4.1. Wie haben Ihnen die Inhalte der App gefallen?

4.2. Zu den Fragebögen: Wie fanden Sie die Bedienung? Wie sind Sie mit dem

Ausfüllen zurechtgekommen?

4.3. Wie könnte man Sie noch mehr motivieren, Fragebögen auszufüllen?

4.4. Zu den Ratgeber-Kapiteln: Wie haben Ihnen diese gefallen?

4.5. Waren die Informationstexte für Sie verständlich?

4.6. Wie angemessen fanden Sie die Ausführlichkeit der Texte?

4.7. Wie zufrieden waren Sie mit der Qualität der Texte?

4.8. Wie empfanden Sie das Themenangebot? … Haben Ihnen Themen gefehlt? Wenn

ja, welche?

4.9. Wie schätzen Sie Ihren Wissenszuwachs durch die Informationstexte ein?

4.10. Zu den Videos: Wie hilfreich fanden Sie die Lehrvideos? …für das Verständnis

der Sachverhalte?

4.11. Was hat Ihnen gefallen? / …Was würden Sie gerne ändern?

4.12. Zu dem individuellen Careplan: Wie haben Sie den Careplan, also die auf Sie

zugeschnittenen App-Inhalte (Termine etc.) wahrgenommen?

4.13. Wie fanden Sie die inhaltliche Zusammenstellung des Careplans?

4.14. Zur Vernetzung mit den Gesundheitsdienstleistern (Health Services): Wie haben

Sie die Integration der verschiedenen Gesundheitsdienstleister wahrgenommen?

4.15. Sind die Informationen zu den verschiedenen Anbietern hilfreich?

4.16. Sind Sie auf einen neuen Anbieter gestoßen, den Sie vorher noch nicht kannten?

4.17. Hat die Nutzung der App Sie motiviert, diese zu kontaktieren?

4.18. Wie könnte man Ihrer Meinung nach den Zugang zu den verschiedenen

Gesundheitsdienstleistern erleichtern?

5. Motivation/Empfehlung

5.1. Würden Sie die App im Verlauf der restlichen Schwangerschaft nutzen?

5.1.1. Wenn nein: Was sind Ihre persönlichen Gründe? Haben Sie Kritikpunkte? Haben Sie Verbesserungsvorschläge?

5.1.2. Wenn ja: Worin sehen Sie den größten Mehrwert?

5.2. Hat sich Ihr Empfinden durch die Nutzung der App verändert? Inwiefern?

5.3. Denken Sie, dass es für andere Patientinnen einen Bedarf für eine App wie

„Mamaherz“ gibt? Wenn Nein: Wieso nicht?

5.4. Würden Sie „Mamaherz“ anderen Schwangeren weiterempfehlen?

5.5. Welche zukünftigen Entwicklungen, im Zusammenhang mit der App

Mamaherz, sind Ihrer Meinung nach wünschenswert?

5.6. Gibt es abschließend noch etwas, das Sie uns mit auf den Weg geben möchten?

Oder etwas, das noch nicht zur Sprache gekommen ist und Sie gerne ergänzen

möchten?

# Anhang 2: System Usability Scale – Deutsche Version, angepasst

Bewertung der folgenden zehn Items jeweils auf einer 5-stufigen Likert Skala von 0 (stimme überhaupt nicht zu) bis 4 (Stimme voll zu).

1. Ich denke, dass ich die Mamaherz App gerne häufig benutzen würde.

2. Ich fand die Mamaherz App unnötig komplex.

3. Ich fand die Mamaherz App einfach zu benutzen.

4. Ich glaube, ich würde die Hilfe einer technisch versierten Person benötigen, um das System benutzen zu können.

5. Ich fand, die verschiedenen Funktionen in diesem System waren gut integriert.

6. Ich denke, die Mamaherz App enthält zu viele Inkonsistenzen.

7. Ich kann mir vorstellen, dass die meisten Menschen den Umgang mit der Mamaherz App sehr schnell lernen.

8. Ich fand die Mamaherz App sehr umständlich zu nutzen.

9. Ich fühlte mich bei der Benutzung der Mamaherz App sehr sicher.

10. Ich musste eine Menge lernen, bevor ich anfangen konnte die Mamaherz App zu verwenden.

**Auswertung:** Die Items 1,3,5,7 und 9 werden mit 0-4 Punkten versehen. Die Items 2,4,6,8 und 10 invertiert mit 0-4 Punkten versehen. Die Gesamtsumme wird mit 2,5 multipliziert und ergibt einen Score zwischen 0-100.

# Appendix 1: Interview guide

The interview focused on the following topics: handling, presentation, content, assessment of the app (motivation, recommendation, added value). Sub-points marked in bold served as guiding questions and were explored in greater depth through other questions during the interview. Where necessary, questions were adapted during the interview or additional topics were added.

1. Introduction before the interview

2. Handling, if poor or not good: Why not?

2.1. How did you generally get on with the MamaHerz app? Were there any difficulties with its general handling?

2.2. In your opinion, what could make the app easier to use?

2.3. Did you encounter any technical problems?

2.4. How did you find the navigation? (*Scrolling and tapping)?*

2.5. Were you able to find the content you were interested in quickly and easily? How clear did you find the presentation of the content?

2.6. Would you like more information or instructions on how to use the app?

3. Presentation

3.1. How did you like the graphic design of the portal?

3.2. ... What would you like to change? / What did you particularly like?

3.3. How did you find the font and icon size used?

3.4. How did you find being addressed informally? Would you have preferred to be addressed formally?

4. Content

4.1. How did you like the content of the app?

4.2. Regarding the questionnaires: How did you find the user interface? How did you find

filling them out?

4.3. How could you be motivated even more to fill out questionnaires?

4.4. Regarding the advice sections: How did you like them?

4.5. Did you find the informational texts easy to understand?

4.6. How appropriate did you find the level of detail in the texts?

4.7. How satisfied were you with the quality of the texts?

4.8. What did you think of the range of topics? ... Were there any topics you felt were missing? If so, which ones?

4.9. How would you rate the increase in your knowledge as a result of the informational texts?

4.10. Regarding the videos: How helpful did you find the instructional videos? ...for understanding the facts?

4.11. What did you like? / ...What would you like to change?

4.12. Regarding the individual care plan: How did you perceive the care plan, i.e., the app content tailored to you (appointments, etc.)?

4.13. How did you find the content compilation of the care plan?

4.14. Regarding networking with health service providers: How did you perceive the integration of the various health service providers?

4.15. Is the information about the various providers helpful?

4.16. Have you come across a new provider that you didn't know about before?

4.17. Has using the app motivated you to contact them?

4.18. In your opinion, how could access to the various healthcare providers be made easier?

5. Motivation/Recommendation

5.1. Would you use the app for the rest of your pregnancy?

5.1.1. If no: What are your personal reasons? Do you have any criticisms? Do you have any suggestions for improvement?

5.1.2. If yes: What do you see as the greatest added value?

5.2. Has your perception changed as a result of using the app? In what way?

5.3. Do you think there is a need for an app like “Mamaherz” for other patients? If no: Why not?

5.4. Would you recommend “Mamaherz” to other pregnant women?

5.5. What future developments related to the Mamaherz app

do you think would be desirable?

5.6. Finally, is there anything else you would like to share with us?

Or is there anything that has not yet been mentioned that you would like to add?

# Appendix 2: System Usability Scale – German version, adapted

Rate the following ten items on a 5-point Likert scale from 0 (strongly disagree) to 4 (strongly agree).

1. I think I would like to use the Mamaherz app frequently.

2. I found the Mamaherz app unnecessarily complex.

3. I thought the Mamaherz app was easy to use.

4. I think I would need the support of a technical person to use this system.

5. I found the various functions in this system were well integrated.

6. I thought there was too much inconsistency in the Mamaherz app.

7. I imagine that most people would learn to use the Mamaherz app very quickly.

8. I found the Mamaherz app very akward to use.

9. I felt very confident using the Mamaherz app.

10. I needed to learn a lot of things before I could get going with the Mamaherz app.

Evaluation: Items 1, 3, 5, 7, and 9 are scored with 0-4 points. Items 2, 4, 6, 8, and 10 are scored inversely with 0-4 points. The total sum is multiplied by 2.5 to give a score between 0 and 100.
